# Stereotactic Body Radiotherapy as Primary Therapy for Head and Neck Cancer in the Elderly or Patients with Poor Performance

**DOI:** 10.3389/fonc.2014.00274

**Published:** 2014-10-08

**Authors:** Arya Amini, Jessica D. McDermott, Gregory Gan, Shilpa Bhatia, Whitney Sumner, Christine M. Fisher, Antonio Jimeno, Daniel W. Bowles, David Raben, Sana D. Karam

**Affiliations:** ^1^Department of Radiation Oncology, University of Colorado School of Medicine, Aurora, CO, USA; ^2^Division of Medical Oncology, Department of Medicine, University of Colorado School of Medicine, Aurora, CO, USA

**Keywords:** stereotactic body radiotherapy, elderly, poor KPS, head and neck cancer

## Abstract

**Objective:** Stereotactic body radiotherapy (SBRT) is increasingly used to treat a variety of tumors, including head and neck squamous cell carcinoma (HNSCC) in the recurrent setting. While there are published data for re-irradiation using SBRT for HNSCC, there are limited data supporting its use as upfront treatment for locally advanced disease.

**Study Design/Methods:** Here, we describe three patients who received SBRT as the primary treatment for their HNSCC along with a review of the current literature and discussion of future pathways.

**Results:** The three cases discussed tolerated treatment well with manageable acute toxicities and had either a clinical or radiographic complete response to therapy.

**Conclusion:** Head and neck squamous cell carcinoma presents a unique challenge in the elderly, where medical comorbidities make it difficult to tolerate conventional radiation, often given with a systemic sensitizer. For these individuals, providing a shortened course using SBRT may offer an effective alternative.

## Introduction

The annual incidence of head and neck squamous cell carcinoma (HNSCC) in the United States is estimated to be around 40,000 (1). While the majority of HNSCC cases occur in the fifth and sixth decade of life, nearly one quarter of patients are older than 70 years of age (2). These tumors predominantly involve the oral cavity and oropharynx with the incidence of both increasing in the United States and worldwide due to the human papillomavirus (HPV) (3, 4). While age may not specifically predict worse disease-specific survival for head and neck cancer patients, the presence of multiple medical comorbidities is known to decrease overall survival rates for these patients (5). HNSCC treatment continues to be a multidisciplinary approach using surgery, chemotherapy, and radiation. While surgery may be an option for some early stage head and neck tumors, the morbidity associated with prolonged surgeries and/or the post-operative functional or physical deformities can be quite detrimental in the elderly (6). Patients with more advanced stage cancers or those not amenable to surgery would typically receive radiation with or without chemotherapy (7–9). Because toxicity is higher with the addition of chemotherapy, combined modality therapy in patients with multiple medical illnesses places them at higher risk of treatment intolerance, which may lead to hospitalizations and treatment interruptions (10). The most commonly used radiation treatment regimen in elderly patients continues to be conventional fractionation of 180–200 cGy per fraction to a total dose of 7000 cGy. Several studies have demonstrated radiation treatment to be quite tolerable in the elderly population with high performance scores (11, 12). When treating elderly patients with multiple comorbidities or dementia, however, life expectancy and performance status along with social issues become important factors that must be weighed into the treatment decision making process.

Given the difficulty of standard HNSCC radiation treatment in elderly individuals with poor performance scores, other treatment options should be considered. Stereotactic body radiotherapy (SBRT) provides an alternative approach for selected patients. This technique can be effective, convenient, and tolerable so long as normal tissue tolerance guidelines are adhered to patients (13). SBRT relies on three fundamental principles: (1) precise, reproducible stereotactic localization of the tumor (either using internal or external references); (2) daily image guidance for tumor re-localization as well as visualization of critical normal organs; and (3) delivered treatment in 1–5 fractions (14). Fractionated SBRT allows for delivery of highly conformal treatment of targets that are in close proximity to critical structures. Fractionation has been hypothesized to improve the therapeutic ratio, thereby reducing the risk of late complications potentially associated with a large single dose (15). The use of non-homogeneity to selectively vary the dose at different sites within the target is another added benefit of hypofractionated radiosurgery as it provides the flexibility to steer a hot spot to the desired target and away from critical structures such as the mandible while treating previously irradiated parotid tumors (15). In other words, a steeper dose gradient is constructed to answer the clinical need. For these reasons, SBRT may be beneficial in elderly patients with multiple comorbidities who would not otherwise tolerate conventional fractionation for head and neck tumors. Here, we present three cases of elderly patients with multiple comorbidities with HNSCC treated primarily with SBRT (Table 1).

**Table 1:** **Patient and treatment characteristics**.

Characteristics	Case #1	Case #2	Case #3
Age	82	72	88
Primary location	Inferior Lip	Left level II/III LN	BOT and left ipsilateral LNs
Total dose (cGy)	3000	2500	3600
Dose per fraction (cGy)	600	500	720
Number of fractions	5 (daily)	5 (daily)	5 (twice-weekly)
Tumor volume (cm^3^)	21.1	36.7	15
Follow-up time (months)	4	8	8
Local control	Near CR (clinical)	PR (clinical)	CR (radiographic)
Toxicity	Grade 2 dermatitis, Grade 1 fatigue	None	Grade 1 mucositis, Grade 1 dermatitis, Grade 2 dysphagia

*CR, complete response; PR, partial response; LN, lymph node*.

## Background

### Case 1

Our first case was an 82-year-old man with multiple medical comorbidities including severe dementia, chronic obstructive pulmonary disease, and type II diabetes, who presented with an enlarging, exophytic mass extending from his lip. He was a former 50 pack year smoker with a long history of daily chewing tobacco use. The lesion presented 6 months prior and homeopathic remedies were attempted prior to presenting to the clinic. On exam, he had a fungating lesion over 40 mm in size involving the central lower lip, sparing the bilateral commissures. The mass extended from the buccal mucosa with no obvious bony involvement. A computed tomography (CT) scan and magnetic resonance imaging (MRI) of the head and neck demonstrated a 37 mm exophytic mass, arising from the midline and left paramedian inner, lower lip with no underlying bony involvement. Biopsy of the mass was positive for ulcerated, invasive, well-differentiated squamous cell carcinoma. It was not tested for HPV. He was staged as T2N0M0 (stage III). He was initially evaluated for a surgical resection and reconstruction expected to last 12 h, but given the high perioperative risks involved, he was determined not to be a surgical candidate. He was therefore referred to radiation oncology for treatment.

**Figure 1:**
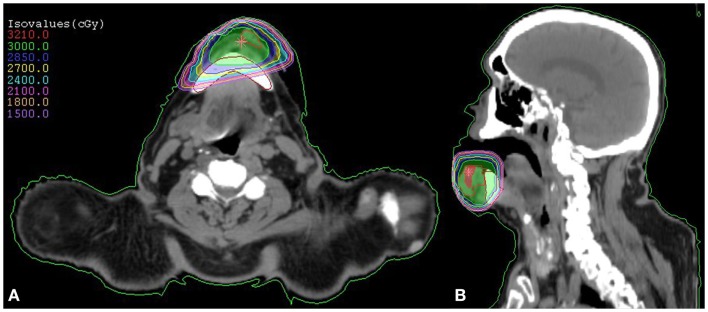
**SBRT dose plan for our patient with squamous cell carcinoma of the lower lip demonstrated by an axial (A) and coronal (B) view**. The prescribed treatment dose of 3000 cGy is demonstrated in green.

**Figure 2:**
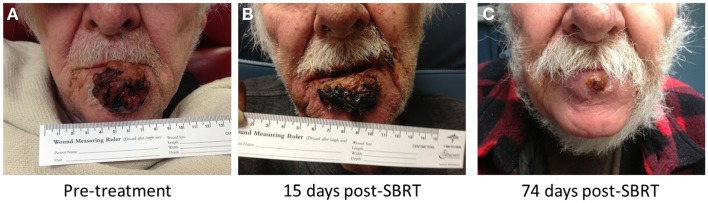
**Our patient with squamous cell carcinoma involving the lower lip, before treatment (A), 15 days (B), and 74 days (C) post-treatment**.

Radiation treatment options were discussed, including intensity modulated radiation treatment (IMRT) given over 6–7 weeks covering his primary and draining lymphatics, versus localized SBRT in five treatments. The patient and his family opted to proceed with SBRT and he received 3000 cGy in five twice-weekly treatments (600 cGy per treatment), with concurrent cetuximab (a loading dose of 400 mg/m^2^ preceding SBRT followed by six weekly infusions of 250 mg/m^2^). The treatment field included the lower lip and buccal mucosa (Figure 1). During treatment, he had noticeable clinical response (Figures 2A,B). He tolerated treatment well with the only adverse effects being grade 2 dermatitis at the treatment site and grade 1 fatigue. He was seen at 2 months follow-up and had a marked improvement in tumor volume and complete resolution of the treatment-related skin erythema (Figure 2C). He had no oral functional deficits after radiation treatment and was satisfied with the cosmetic outcomes. At the time of manuscript submission, he was 12 months out from treatment with continued response and no evidence of toxicity.

### Case 2

Our second case was a 72-year-old man with multiple comorbidities who initially presented to his primary care physician after his wife noticed an enlarging, painful left neck mass. His past medical history was significant for severe dementia requiring hospitalizations, bradycardia requiring a pacemaker, carotid artery disease, and hypertension. He was a non-smoker who drank alcohol occasionally. Imaging that included a CT scan identified an enlarging left cervical lymph node with central necrosis, measuring 3 cm. Fine needle aspiration (FNA) of the lymph node was positive for squamous cell carcinoma (HPV testing not performed). Flexible nasopharyngoscopy could not identify the primary site of disease. A follow-up PET scan again identified a 33 mm × 30 mm left level II lymph node, standardized uptake value (SUV) 12.5, and a 25 mm × 13 mm right level II lymph node, SUV 4.3. There were no other areas of FDG avidity. He was staged cT0N2cM0 (stage IVA) and was referred to radiation oncology to discuss treatment options. At the time of presentation, he was in an acute rehabilitation facility for progressive dementia and antibiotics for a recent bacteremia.

Given his severe dementia, it was concluded he would not tolerate standard head and neck treatment. Further workup, including directed biopsies and tonsillectomy, was also declined given his high perioperative risks. Therefore, he was treated with SBRT to 2500 cGy in five treatments given daily (500 cGy per treatment), with no concurrent systemic sensitizer. The treatment field included the enlarging left cervical lymph node encompassing levels II/III, which was limiting his head movements. During treatment, he had some response in the left neck with resolution of the palpable lymph node. He did not develop any notable toxicity from treatment, including dermatitis, mucositis, or esophagitis. The plan was to return and treat the right cervical lymph node as well, however, his dementia rapidly progressed following treatment and he soon entered hospice care. He passed away 8 months after completing treatment from causes unrelated to his cancer. At that time, he had no clinical evidence of disease at the treated left cervical node.

### Case 3

Our third case was an 88-year-old woman who presented with a painful left neck mass for 1 month with associated weight loss. She was a non-smoker with no significant past medical history. PET scan identified a large hypermetabolic left cervical lymph node, measuring 44 mm × 29 mm (SUV 12.2), a 9 mm left cervical node (SUV 7.0) with asymmetry at the left base of tongue. Incidentally, a hypermetabolic 15 mm left breast lesion was also found, along with left axillary and subpectoral lymphadenopathy. There was also FDG avidity involving the fifth lumbar (L5) vertebral body, with an associated destructive lesion. FNA of the left cervical mass was positive for squamous cell carcinoma, HPV positive by p16 staining. Breast biopsy was consistent with intraductal carcinoma (ER/PR positive, HER2/neu negative) and she was staged T1cN1M0 (stage IIA). Biopsy of the L5 lesion was consistent with poorly differentiated carcinoma, pathologically similar to the biopsied cervical lymph node. She was staged as cT1N2bM1 (stage IVC), base of tongue primary.

Given the systemic involvement of her HNSCC, her concurrent breast cancer, and patient refusal for a prolonged course of radiation treatment, SBRT was offered for local and symptomatic control. She underwent radiation treatment with SBRT, treated to 3600 cGy in five twice-weekly treatments (720 cGy per treatment) to gross disease including base of tongue and 3000 cGy in five twice-weekly treatments (600 cGy per treatment) to ipsilateral, uninvolved draining lymph nodes. During treatment, she developed some initial mild left neck swelling which quickly resolved. She also experienced grade 1 mucositis, grade 1 dermatitis, oral thrush, and grade 2 dysphagia toward the end of treatment. Following completion of treatment to her head and neck, her L5 vertebral body was treated with SBRT, 2700 cGy in three twice-weekly treatments (900 cGy per treatment). She elected to not receive any treatment for her breast cancer. At 4 month follow-up, her treatment-related side effects had resolved and she clinically had no evidence of disease in her head and neck, though multiple new hypermetabolic lesions were found in the right femoral neck, gastric fundus, and right hepatic lobe. These were not biopsied to differentiate between metastatic head and neck versus metastatic breast cancer. She received palliative treatment to her right femur and L4-S1 vertebral bodies, 2000 cGy in five treatments given every other day (400 cGy per treatment). Repeat PET scan at 6 months showed further progression of disease including multiple new liver lesions, bone lesions involving the spine and ribs, pancreatic mass, and peritoneal carcinomatosis. The left cervical lymph node conglomeration had decreased in size and FDG avidity, and no evidence of disease was observed at the left base of tongue. The patient passed away 8 months after original diagnosis due to her metastatic disease.

## Discussion

For elderly patients with HNSCC or in younger patients with poor performance status, proper assessment of their medical conditions is critical in the initial workup. While elderly patients with good performance status should receive standard of care (12), those with multiple comorbidities who cannot tolerate standard therapy may benefit from a shortened, local consolidative treatment approach. Although definitive chemoradiation is associated with improved overall survival benefit (9), it comes at a price of substantial morbidity in a patient population with baseline multiple medical comorbidities due to the often long-term use of tobacco and excessive alcohol consumption (10, 16–18). This may suggest why some elderly patients perhaps have less benefit to treatment (9), as they present with multiple medical issues, which can lead to poorer treatment compliance (10, 18).

Currently, there is growing literature supporting the use of both conventional hypofractionated external beam radiotherapy and higher dose per fraction SBRT for primary or recurrent head and neck treatment in patients who are inoperable and cannot tolerate conventional fractionation (19, 20). Two small Australian studies evaluated hypofractionated palliative radiation as primary treatment for incurable or medically unsuitable patients. The first, “QUAD SHOT,” consisted of 1400 cGy in four fractions given twice a day for two days and then repeated up to two more times at 4-week intervals if no tumor progression occurred. In all, 53% had an objective response and 23% had stable disease with overall survival of 5.7 months (21). The other study, “Hypo-Trial,” gave 3000 cGy in five fractions at two fractions/week. The overall objective response rate was 80% and median time to death was 6.1 months (22). Both studies prospectively assessed quality of life during treatment [using either the EORTC QLQ-C30 or Functional Assessment of Cancer Therapy (FACT) methods], and both showed improvement in quality of life parameters. In addition, a number of studies published have reported outcomes with SBRT in both the upfront and recurrent setting (Table 2). A small, retrospective series recently published from Japan (23) reviewed 14 elderly patients who received primary SBRT without a sensitizer for the initial management of their head and neck cancers. Radiation doses ranged from 3500 to 4200 cGy, given in 3–5 fractions. At a mean follow-up of 3 years, local control and overall survival were 71.4 and 78.6%, respectively. Toxicities were mostly grade 1 or 2 with one grade 3 osteonecrosis in a patient who received a second treatment of SBRT following disease recurrence. Similarly, in another retrospective analysis of elderly patients treated with primary SBRT for salivary gland tumors, Karam et al. showed 2-year local control rate of 84% at a median follow-up of 14 months (24). The treatment was also reportedly well tolerated with no grade 4 toxicities. Lastly, a series evaluating recurrent nasopharyngeal carcinoma also demonstrated favorable outcomes in the SBRT group when compared to conventional fractionation (25).

**Table 2:** **Review of SBRT for head and neck cancers**.

Authors (reference)	Prospective/retrospective study	Number of patients	First-line or recurrent therapy	Radiation course	Concurrent therapy	Median PFS	Median OS
Heron et al. (13)	Prospective	25	Recurrent	25–44 Gy total in 5 fractions over 2 weeks	N/a	4 mo	6 mo
Roh et al. (19)	Retrospective	36	Recurrent	18–40 Gy in 3–5 fractions	N/a	61% at 12 mo	16.2 mo
Siddiqui et al. (20)	Retrospective	44	Both	Range of single fraction 13–18 Gy or 36–48 Gy in 5–8 fractions	N/a	83.3% at 12 mo (primary), 60.6% at 12 mo (recurrent)	28.7 mo (primary), 6.7 mo (recurrent), 5.6 mo (metastatic)
Kawaguchi et al. (23)	Retrospective	14	1st line	35–42 Gy in 3 or 5 fractions	S-1 (an oral 5-fluorouracil)	71.4% at 36 mo	78.6% at 36 mo
Rwigema et al. (26)	Retrospective	85	Recurrent	Median dose 35 Gy in fraction sizes of 4–18Gy	N/a	5.5 mo	11.5 mo

*PFS, progression free survival, OS, overall survival, mo: months*.

Stereotactic body radiotherapy also represents a more convenient and cost-effective approach of treating elderly patients with poor performance status. At times in our experience, patients must travel long distances and it may be a burden financially for these patients. Some elderly patients at our center have to travel long distances for treatment and may not have the social support or financial means to stay away from home for 6–7 weeks and simply refuse treatment if it cannot be offered over a shorter time period. In fact, we have also encountered this situation in Colorado with patients less than 70 years of age with excellent performance status. SBRT offers a rapid and precise alternative strategy for these individuals with poor prognostic scores and locoregionally confined disease through the use of improved imaging modalities, implementation of sophisticated planning, and delivery systems with daily image guidance (27). Lastly, when evaluating radiation treatment modalities used in other disease sites, SBRT has been shown to be very cost-effective (28–30).

Radiobiologically, the higher dose per fraction with SBRT-based treatments has been shown to provide improved local control over standard fractionation. As the survival and proliferation of tumor cells are directly dependent on the blood supply, SBRT has been shown to have a direct effect on tumor vasculature. High-dose radiation with 10 Gy or higher in a single fraction has been shown to cause severe vascular damage in human tumor xenografts or animal tumors (31, 32). Additionally, the vascular injury and ensuing chaotic intratumor environment, such as hypoxic, acidic, and nutritionally deprived environment caused by high-dose fraction SBRT, may significantly hinder the repair of radiation damage (33). However, one must still remain cognizant of neighboring critical structures and as such, our patients did not receive fractions of 10 Gy or higher.

Dose constraints in the setting of primary SBRT for head and neck cancer are extrapolated from the head and neck re-irradiation literature and from other systems as data for constraints in the primary setting are lacking. In lieu of this, we have attempted to draw from the Quantitative Analysis of Normal Tissue Effects in the Clinic (QUANTEC), head/neck re-irradiation literature and clinical studies to help guide individuals interested in pursuing head/neck SBRT. In the primary setting, spinal cord SBRT dose constraints are the most studied and documented. Per published QUANTEC guidelines, spinal SBRT partial cord irradiation max dose constraints is reported at 13 Gy for single fraction treatment and 20 Gy for three fractions treatment is thought to be associated with <1% risk for myelopathy (34). Based on our own institutional experience combined with Dr. Timmerman at UT Southwestern, constraints for five fractions are more generous allowing for a max point of 28 Gy and V22 < 10% assuming 5–6 mm above and below the spinal cord subvolume being treated (unpublished data). Typical re-irradiation dose constraints derived from the Pittsburgh and Georgetown series (26, 35) tend to be more conservative (spinal max point ≤ 8 Gy in one fraction and ≤ 12 Gy in two fractions) but again, these are based on re-irradiation SBRT compared to the established 10 Gy to 10% of partial spinal cord being irradiated in the upfront setting (36). Similarly for brainstem, *D*_max_ < 12.5 Gy in a single fraction is predicted to be associated with <5% risk for cranial neuropathy or necrosis (37). The NRG head and neck committee is currently developing an SBRT trial for recurrent HNC that will evaluate its efficacy and safety in combination with immunomodulation using a PD-1 antibody.

In addition to the present limitations of current data on SBRT toxicity for head and neck cancers as discussed, the first two cases demonstrate the challenge of treating patients with dementia. SBRT relies on reproducibility, which may be difficult to maintain in patients who are unable to remain still. Additionally, patients with dementia require redirecting and daily coaching in order to tolerate and complete radiation therapy. Given the morbidity associated with untreated head and neck cancers, however, it is still reasonable to treat head and neck cancer patients with dementia and as shown in the first two cases, a shortened course of radiation may be better tolerated and more manageable than a standard course of therapy. Ultimately, a lengthy discussion is indicated between the radiation oncologist, patient, and family to assess tolerability of treatment.

For other head and neck sites, our recommendations derive from the re-irradiation literature and some prospective studies. However, assuming SBRT in the primary setting, dose constraints are likely to be more generous given lack of prior radiotherapy but we would caution a more conservative approach combined with clinician judgment in the absence of any prospective data.

## Concluding Remarks

Management of elderly patients with HNSCC who present with multiple comorbidities can pose a unique challenge. SBRT therefore may be a viable option for elderly patients unable to receive standard of care combined modality therapy. Of the available radiation treatments, however, SBRT has arguably the greatest potential for benefit and harm due to the very high, ablative doses of radiation used. This approach therefore warrants a prospective study and may be especially appropriate for well-lateralized head and neck cancers. In addition, incorporation of biologically based agents such as EGFR inhibitors, DNA repair inhibitors, or immunomodulation may enhance local-regional effectiveness of SBRT without a significant increase in acute toxicity.

## Conflict of Interest Statement

The authors declare that the research was conducted in the absence of any commercial or financial relationships that could be construed as a potential conflict of interest.
